# The Relationship Between Neutrophil-to-Lymphocyte Ratio, Platelet-to-Lymphocyte Ratio, and Systemic Immune-Inflammation Index Markers and Response to Biological Therapy in Patients with Psoriasis

**DOI:** 10.3390/ijms26083868

**Published:** 2025-04-19

**Authors:** Agnieszka Kimak-Pielas, Ewa Robak, Radosław Zajdel, Agnieszka Żebrowska

**Affiliations:** 1 Department of Dermatology and Venereology, Teaching Hospital No 2, 90-549 Lodz, Poland; 2 Department of Dermatology, Medical University of Lodz, 90-647 Lodz, Poland; 3 Department of Economic and Medical Informatics, University of Lodz, 90-214 Lodz, Poland; 4 Department of Medical Informatics and Statistics, Medical University of Lodz, 90-645 Lodz, Poland

**Keywords:** psoriasis, biological therapy, neutrophil-to-lymphocyte ratio, platelet-to-lymphocyte ratio, systemic immune-inflammation index

## Abstract

Plaque psoriasis is a chronic, immune-mediated inflammatory skin disease characterized by the formation of thick, scaly plaques. The disease is driven by dysregulation of the immune response, primarily involving T-helper cells, which create a persistent inflammatory environment. In recent years, several biomarkers reflecting systemic inflammation have been identified, including indices derived from a complete blood count, such as the neutrophil-to-lymphocyte ratio (NLR), platelet-to-lymphocyte ratio (PLR), and Systemic Immune-Inflammation Index (SII). The aim of our study was to explore the role of these markers in patients with psoriasis undergoing biological treatment. Medical records of 159 patients with plaque psoriasis receiving biologics were retrospectively reviewed. The NLR, PLR, and SII values were calculated from the hemograms of the patients. Additionally, demographic and psoriasis severity data were analyzed. During the 18-month follow-up, the mean NLR, PLR, SII, and CRP values were significantly decreased in comparison to the baseline (*p* < 0.05). No significant differences between anti-TNF, anti-IL-12/23, anti-IL-17, and anti-IL-23 drugs were identified (*p* > 0.05). The initial values of NLR, PLR, and SII were positively correlated with psoriasis severity. No relationship between the analyzed biomarkers and age, sex, psoriasis duration, and prior exposure to biological drugs was identified. CBC-derived biomarkers may be useful for monitoring inflammation reduction in psoriasis patients treated with biological drugs.

## 1. Introduction

Psoriasis (PsO) is a chronic autoimmune disease affecting about 2–3% of the population. The disease is characterized by erythematous papules and plaques covered with silvery scales, and affects predominantly elbows, knees, and scalp [1]. PsO is primarily caused by an interplay between genetic, environmental, and immune factors. The immunologic pathways particularly involve Th1 cells and pro-inflammatory cytokines, including tumor necrosis factor-alpha (TNF), interleukin 17 (IL-17), and interleukin 23 (IL-23) [2,3]. Treatment strategies vary based on disease severity, from topicals and phototherapy to conventional systemic treatment and biological therapy. Biological drugs include anti-TNF (etanercept, adalimumab, infliximab, certolizumab pegol), anti-IL-17 (ixekizumab, secukinumab, brodalumab, bimekizumab), anti-IL-12/23 (ustekinumab), and anti-IL-23 (guselkumab, tidrakizumab, risankizumab) molecules [2,4].

In recent years, several biomarkers have been identified as indicators of the body’s inflammatory status [5,6,7]. Those derived from routine blood tests are particularly interesting because they are inexpensive, widely available, and easy to perform. Indices derived from complete blood count (CBC), such as the neutrophil-to-lymphocyte ratio (NLR), platelet-to-lymphocyte ratio (PLR), and Systemic Immune-Inflammation Index (SII), are used as versatile markers highlighting an intriguing interplay between the innate immune response (neutrophils), the adaptive immune response (lymphocytes), and platelets, which play a role not only in coagulation but also in the inflammatory response [5,8]. While no universally fixed reference ranges exist, values observed in healthy adult populations are summarized in Table 1 [9]. These values can vary depending on the age, sex, ethnicity, and underlying health conditions and are not usually used as strict thresholds. Instead, they can be useful in monitoring and evaluating treatment effectiveness before clinical improvement becomes apparent.

CBC-derived inflammatory biomarkers are studied in many conditions, including cancer, cardiovascular, autoimmune, and infectious diseases [10,11,12,13,14,15]. However, their utility in psoriasis has not yet been fully determined. In 2019, Paliogiannis et al. conducted a meta-analysis to summarize the role of NLR and PLR in psoriasis, and in 2024, Ye et al. updated it [8,16]. Both groups concluded that NLR and PLR are elevated in patients with psoriasis but are not correlated with disease severity. SII is another hematological derivative that is increased in the psoriasis population compared to healthy volunteers and might even serve as a marker of psoriasis severity [17,18,19]. However, data from the literature on clinical implications and cut-off values in psoriasis are relatively scarce and inconclusive (Table 1) [5,20,21,22,23,24,25,26,27].

While biological drugs have revolutionized psoriasis management, there is no standardized marker to guide a personalized approach in biological treatment. This study aims to address this gap by evaluating the change in NLR, PLR, SII, and CRP (C-reactive protein) in patients with psoriasis undergoing biological treatment and exploring their potential to differentiate between biologic agents.

**Table 1:** A summary of inflammatory biomarkers assessed in the research, based on sex and age.

Index	Measurement Method	Normal Range [9]					Cut-off Values in PsO
NLR	Neutrophils ÷ Lymphocytes	**Males**		**Females**			≥1.66 in PsO patients [25] >2.239 increases the risk of PsA [21] >2.8 increases the risk of PsA [24] >2.63 increases the risk of more severe PsO [20] >2.35 increases the risk of moderate vs. mild PsO [22] >2.11 increases the risk of PASI >10 [26] >2.32 increases the risk of subclinical atherosclerosis [23]
		18–60 yrs old	≥61 yrs old	18–50 yrs old	51–70 yrs old	≥71 yrs old	
		0.86–3.45	0.89–4.27	0.85–3.7	0.79–3.3	0.88–3.86	
PLR	Platelets ÷ Lymphocytes	**Males**		**Females**			≥110.6 in PsO [25] >111.9 increases the risk of PASI >10 [26] >159.6 increases the risk of PsA [21]
		18–70 yrs old	≥71 yrs old	18–70 yrs old		≥71 yrs old	
		62.96–200.6	55.95–216.41	69.67–216.9		57.93–264.64	
SII	(Neutrophils × Platelets) ÷ Lymphocytes	**Males**		**Females**			>408.8 increases the risk of moderate vs. mild PsO [22] >552.9 increases the risk of PASI >10 [26]
		≥18 yrs old		18–50 yrs old	51–60 yrs old	≥61 yrs old	
		190.5–760.9		171.3–998.0	167.04–973.56	146.7–991.3	
CRP	Serum assay	<5 mg/L					≥2 mg/L increases the risk of PsO [27]

yrs—years, PsO—psoriasis, PsA—psoriatic arthritis.

## 2. Results

### 2.1. Patient Characteristics

The analyzed group included 159 patients with a mean age of 44.35 ± 13.94 years at the beginning of the biological treatment. Of this group, 43.40% (69 patients) were women, and 56.60% (90 patients) were men. The mean age of psoriasis onset was 25.73 ± 13.57 years, and the mean duration of the disease before the biological treatment initiation was 18.61 ± 12.96 years. A total of 130 patients were bio-naïve, while the remaining 29 patients had been exposed to biological drugs in the past. In the analyzed group, 62.89% of patients (100) received only one biologic treatment cycle, while the others (59 patients) received multiple cycles due to the primary or secondary lack of effectiveness, administrative discontinuation, or experienced side effects. In addition, 40 patients were enrolled in the program by meeting the criteria related to the involvement of specific areas. Psoriasis severity for a total population was as follows: DLQI (Dermatology Life Quality Index) 19.38 ± 6.06, BSA (Body Surface Area) 23.08 ± 15.2, and PASI (Psoriasis Area and Severity Index) 17.39 ± 8.24, while for the patients with specific areas enrollment, the severity was as follows: DLQI 20.94 ± 6.72, BSA 7.98 ± 5.25, and PASI 7.29 ± 4.07. The variables are summarized in Table 2.

In total, we analyzed 100 drug-periods with anti-IL-23 drugs (8 with tildrakizumab, 69 with risankizumab, 33 with guselkumab), 77 cycles with anti-TNF (68 with adalimumab, 9 with infliximab), 55 drug-periods with anti-IL-17 (22 with ixekizumab, 33 with secukinumab), 20 cycles with bimekizumab, and 38 cycles with ustekinumab.

### 2.2. NLR, PLR, SII, and CRP Changes During the Treatment with Biological Drugs

**Figure 1:**
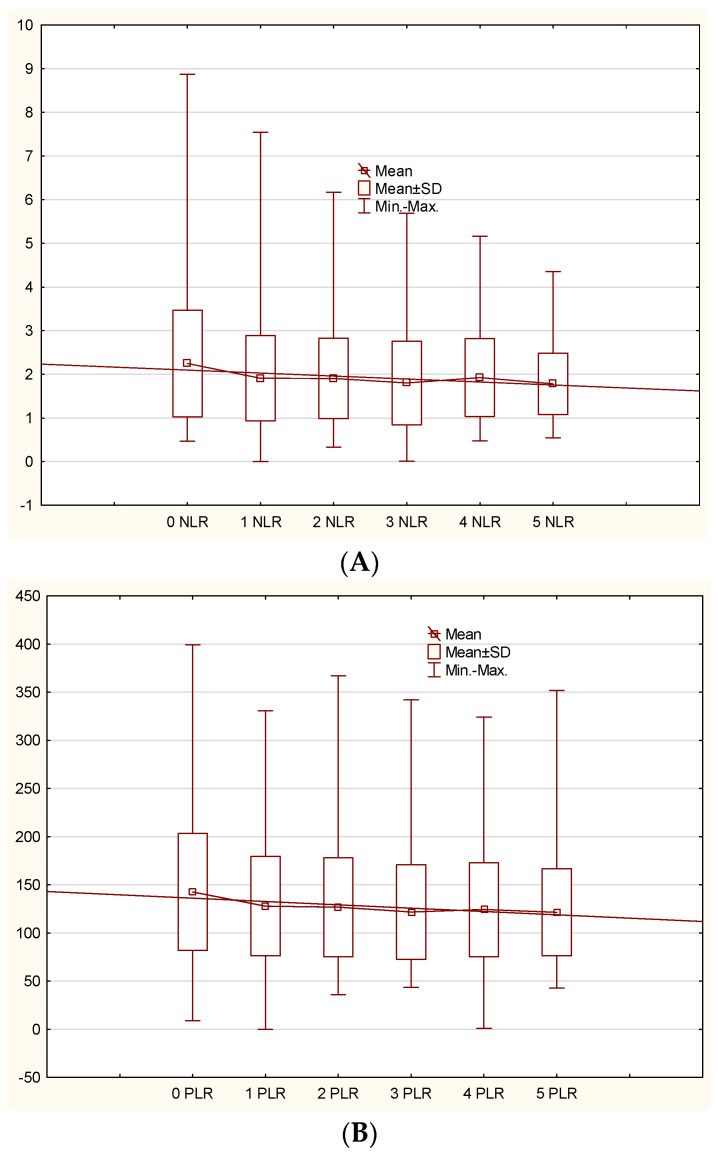

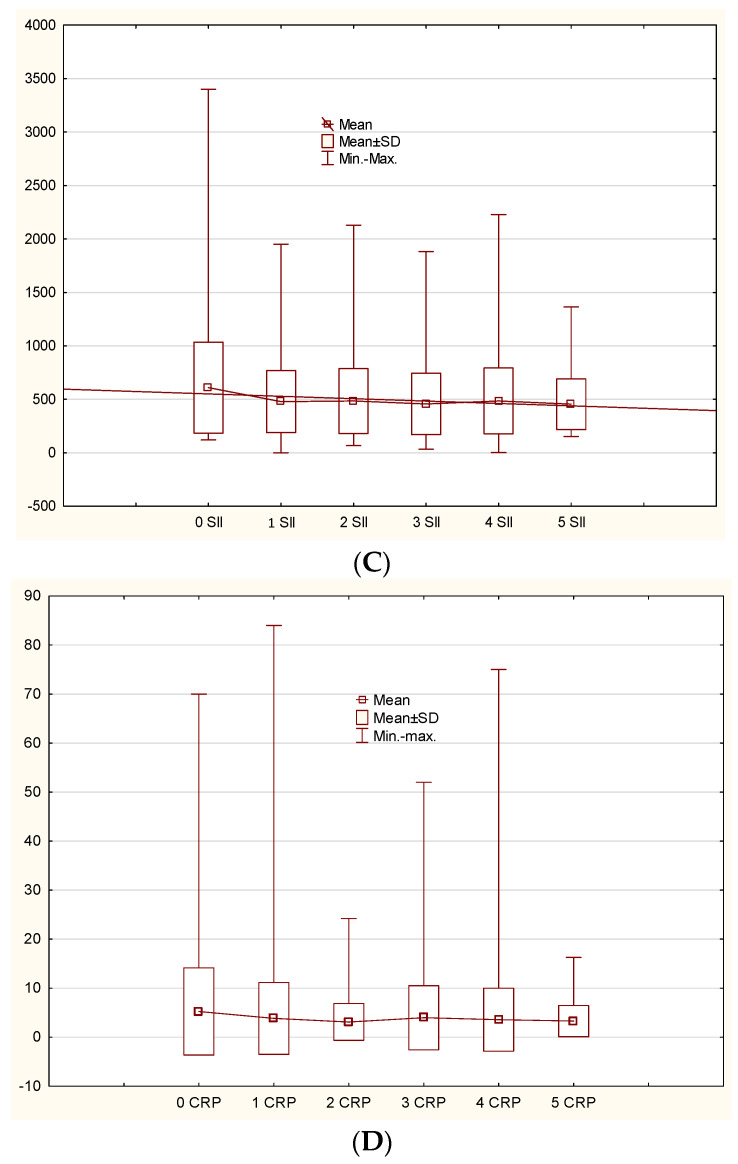
NLR, PLR, SII, and CRP reduction in the patients treated with biological drugs. The reduction between timepoints 0 and 5 was statistically significant, whereas the decrease between timepoints 1 and 5 did not reach statistical significance. (**A**) NLR 0–5 *p* = 0.00335, NLR 1–5 *p* = 0.65496, (**B**) PLR 0–5 *p* = 0.00270, PLR 1–5 *p* = 0.38538, (**C**) SII 0–5 *p* = 0.00001, SII 1–5 *p* = 0.19407, (**D**) CRP 0–5 *p* = 0.00010, CRP 1–5 *p* = 0.70954 (ANOVA).

The analysis of all patients treated with biological drugs revealed significant changes in NLR, PLR, SII, and CRP scores over the observation period, with *p*-values of 0.00335 for NLR, 0.00270 for PLR, 0.00001 for SII, and 0.00010 for CRP (Figure 1). We observed a decrease in NLR value from 2.338021 ± 1.35455 before the treatment to 1.815243 ± 0.759045 after 18 months. Similarly, PLR decreased from 146.0028 ± 59.20787 to 124.9965 ± 44.00531, and SII reduced from 593.6625 ± 389.9643 to 453.8275 ± 251.4009 over the same period. For a total population of patients, the decrease in CRP levels was observed from 5.6571 ± 6.8202 to 2.5600 ± 2.3651 during the 18-month observation.

Interestingly, when excluding the initial values of these parameters, the observed decreases in NLR, PLR, SII, and CRP from visit 1 to visit 5 were not significant (*p* = 0.65496, *p* = 0.38538, *p* = 0.19407, *p* = 0.70954, respectively). The NLR, PLR, SII, and CRP values for a total group of patients and separately for each agent are provided in the Supplementary Materials (Table S1).

### 2.3. NLR, PLR, SII, and CRP Comparison Between Different Therapies

The analysis of NLR, PLR, SII, and CRP changes for each biological agent was conducted, followed by a comparison of the results. In the calculated CBC-derived markers, statistically significant changes were observed only in PLR for anti-TNF, as well as in SII for anti-TNF and anti-IL-17 therapies, with *p*-values of 0.01763, 0.03680, and 0.01455, respectively. Other parameters and drugs showed insignificant results (*p* > 0.05). CRP values were significantly reduced after the administration of TNF inhibitors (*p* = 0.01809) but not after other drugs (*p* > 0.05) (Supplementary Materials, Table S2).

Moreover, no significant differences were found when we directly compared the changes in NLR, PLR, SII, and CRP among patients treated with anti-TNF, anti-IL-23, anti-IL-17, and anti-IL-12/23 drugs (*p* > 0.05). The only exception was the PLR comparison between anti-IL-23 and anti-TNF therapies, where *p* = 0.016956 (ANOVA). Subsequently, the analysis using multivariate tests for repeated measures was conducted for these parameters. Ultimately, Wilks’ Lambda (*p* = 0.214833), along with Pillai’s Trace, Hotelling’s Trace, and Roy’s Largest Root, all yielded non-significant results (*p* > 0.05) (Supplementary Materials, Table S3).

### 2.4. NLR, PLR, and SII Changes in Patients with Specific Areas Enrollment

In the group of patients with involvement of specific areas (nails, hands and feet, scalp, face, or anogenital area), changes in NLR, PLR, and SII did not reach statistical significance (NLR *p* = 0.23324, PLR *p* = 0.12555, SII *p* = 0.30510). Subsequently, we investigated whether the differences between this subgroup and the overall patient population were statistically significant. An analysis using generalized linear modeling and multivariate tests was performed for repeated measures of the NLR, PLR, and SII parameters. Wilks’ test (*p* = 0.721, *p* = 0.858, *p* = 0.739, respectively), along with Pillai’s, Hotelling’s, and Roy’s tests, did not reveal any statistically significant differences (*p* > 0.05) in the variability of successive NLR, PLR, and SII measurements between these two groups. Tukey’s HSD test was also not significant for these parameters (*p* > 0.05), and CRP changes did not change significantly (*p* > 0.05) (Table S1).

### 2.5. Correlation Between NLR, PLR, and SII and Demographic and Clinical Variables

The correlations between NLR, PLR, and SII and different variables were investigated, followed by conducting ANOVA for repeated measures for age, sex, psoriasis duration, initial CRP, or prior exposure to biological drugs. No statistically significant relationship was identified for these parameters (*p* > 0.05).

**Figure 2:**
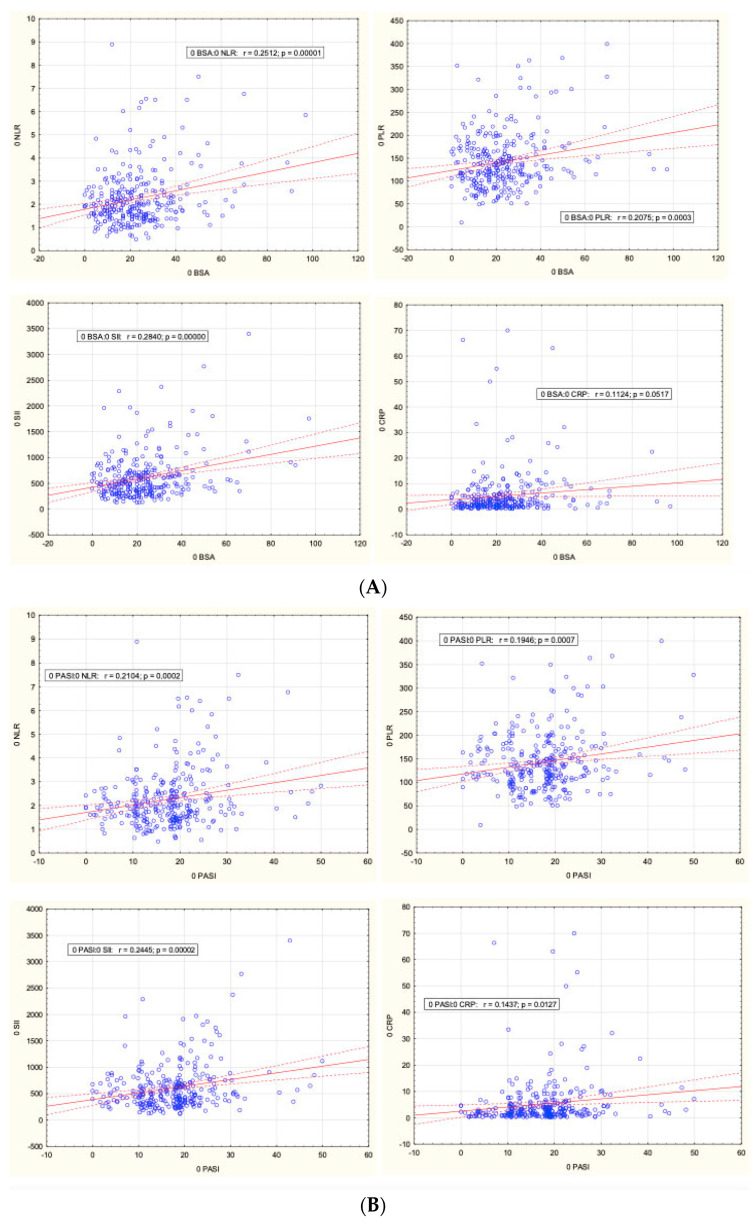
Correlation between NLR, PLR, SII, and CRP and BSA (**A**) and PASI (**B**). Dashed lines represent the 95% confidence interval (CI95).

In contrast, there was a weak positive correlation between CBC-derived biomarkers and psoriasis severity. Linear regression with one variable (BSA or PASI) is presented in Figure 2. The correlation between NLR, PLR, SII, and BSA and PASI, as well as between CRP and PASI, was statistically significant (*p* < 0.05). A correlation between CRP and BSA was close to reaching statistical significance (*p* = 0.05170). The R-Spearman correlation model with multiple variables for baseline BSA (BSA0) and baseline PASI (PASI0) was as follows:

- NLR0 r = 0.1508 (*p* = 0.0089) for BSA0 and r = 0.1572 (*p* = 0.0064) for PASI0,
- PLR0 r = 0.1314 (*p* =0.0228) for BSA0 and r = 0.1069 (*p* = 0.0644) for PASI0,
- SII0 r = 0.1683 (*p* = 0.0035) for BSA0 and r = 0.1712 (*p* = 0.0029) for PASI0,
- CRP0 r = 0.1665 (*p* = 0.0038) for BSA0 and r = 0.1988 (*p* = 0.0005) for PASI0.

### 2.6. Correlation Between NLR, PLR, and SII

**Figure 3:**
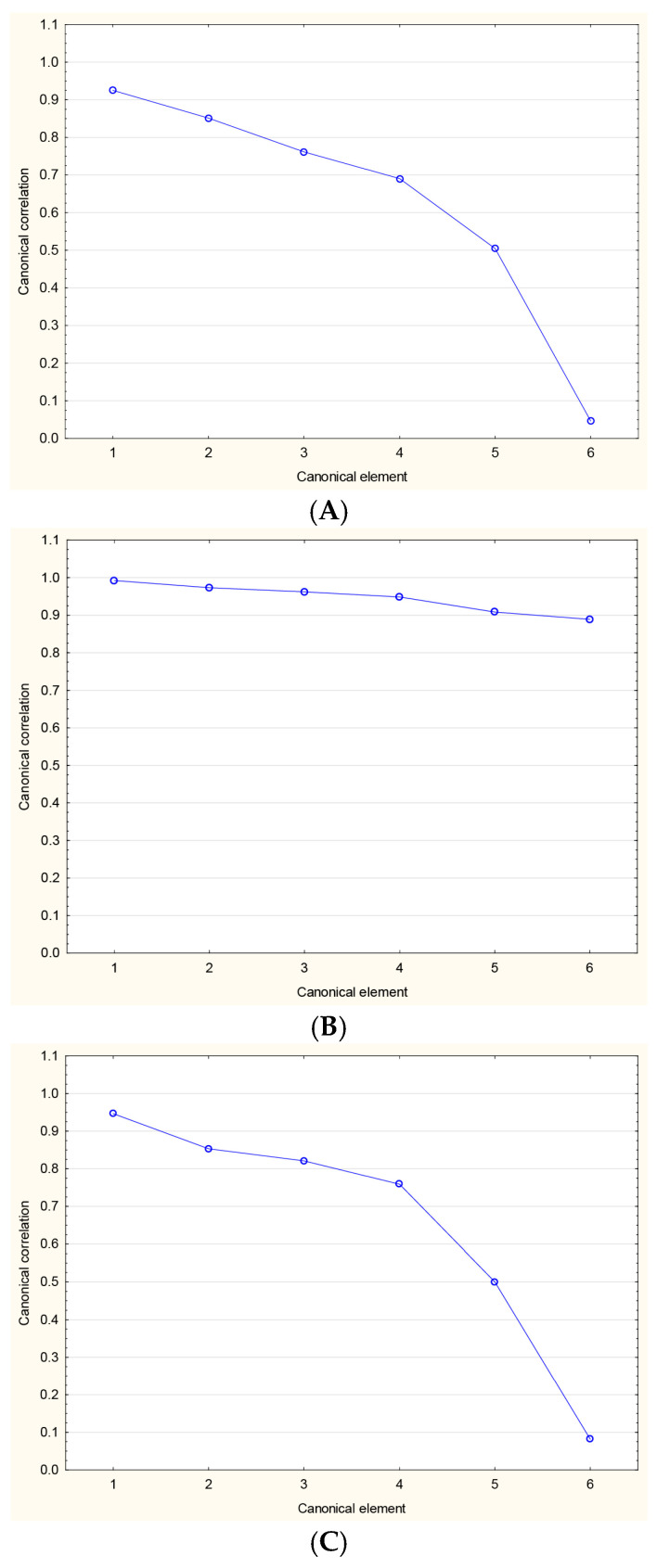
Canonical analysis of correlations between NLR, PLR, and SII changes. (**A**) NLR vs. PLR, (**B**) NLR vs. SII, (**C**) PLR vs. SII.

The NLR, PLR, and SII changes during the study period followed a non-normal distribution. To analyze those multidimensional variables with a non-normal distribution, canonical analysis was implemented. The results are presented in Figure 3. The values are provided in Supplementary Materials (Table S4). A total redundancy of 62.31%, 91.30%, and 70.07%, alongside a statistical significance of *p* < 0.05, indicates a correlation between NLR and PLR; NLR and SII; and PLR and SII, respectively. It is noteworthy that canonical correlations between NLR 4/5 and PLR 4/5, as well as between PLR 4/5 and SII 4/5, were not statistically significant, while for NLR 4/5 and SII 4/5, significance was retained throughout the whole observation period.

## 3. Discussion

Psoriasis is a chronic immune-mediated skin condition driven by a dysregulation of the innate and adaptive immune systems [2]. The evaluation of the degree of inflammation might be challenging. Potential indicators of this intricate interplay between various immunological components include NLR (neutrophil-to-lymphocyte ratio), PLR (platelet-to-lymphocyte ratio), NMR (neutrophil-to-monocyte ratio), MLR (monocyte-to-lymphocyte ratio), and SII (Systemic Immune-Inflammation Index) [8,28,29]. They potentially offer an insight into inflammation severity and allow an objective assessment of the severity. Our research aimed to investigate NLR, PLR, SII, and CRP levels in patients with psoriasis treated with biological drugs. We demonstrated a significant reduction in all these markers during the 18-month follow-up and showed a positive correlation between their values and psoriasis severity. The study presents a promising approach to objectively assessing treatment effectiveness beyond clinical skin examination, offering potential benefits for long-term disease management.

The available research indicates that the NLR, PLR, and SII values decrease during systemic treatment for PsO, and our study supports these findings [28,30,31,32,33,34]. Although in our patients the reduction was significant in the overall population, we did observe a statistical significance for individual agents only when analyzing the change in PLR for anti-TNF and SII for anti-TNF and anti-IL-17 therapies. For other molecules, the decrease was insignificant (*p* > 0.05). A direct comparison between the agents did not reveal any significant differences (*p* > 0.05). The literature comparing CBC-derived biomarkers across different biological drugs is limited and remains inconclusive. For instance, Albayrak evaluated NLR and PLR values in the first six months of treatment with several anti-TNF, anti-IL-17A, and anti-IL-12/23 agents and reported no significant differences among them [28]. In a comparable manner, another study revealed that after treatment with infliximab, adalimumab, and ustekinumab for up to a year, NLR and PLR values decreased swiftly, irrespective of the type of biologics used [30]. Other groups investigating the impact of biologics on CBC-derived parameters concluded similarly [31,32]. In contrast, a Turkish study found that adalimumab therapy resulted in a significant decrease in NLR values, while other examined agents had an insignificant impact on neutrophil-to-lymphocyte ratio. However, it is worth noting that the analyzed group was relatively small, consisting of a total of 35 patients treated with biologics [33]. In addition, significant variations in blood-count-derived inflammatory markers, e.g., NLR for adalimumab, ixekizumab, and secukinumab, were noted in research conducted by Morariu et al. However, as the biologic agents were not directly compared within the study, it remains unclear whether the differences between them are statistically significant [34]. Further research comparing various biological drugs is essential to draw robust conclusions.

It is noteworthy that in our study, the decrease in NLR, PLR, SII, and CRP levels was statistically significant only when the baseline values were included. Without these pre-treatment values, the reduction did not reach statistical significance. This finding highlights the potential importance of the initial weeks of biological treatment, during which the majority of immune modulation may occur. However, this hypothesis requires further validation.

Another parameter explored by our group, C-reactive protein (CRP), is an acute-phase molecule correlated with many conditions and not specific to psoriasis. It may serve as a bioindicator of PsO severity and the presence of PsO comorbidities, especially psoriatic arthritis [27,35]. However, due to its sensitivity and variability between individuals, interpreting CRP levels is challenging and should be performed alongside other biomarkers for a more accurate evaluation [36,37]. Although a correlation between CRP and PASI exists in patients receiving treatment, it appears to be relatively weak [38,39,40,41]. In our study, CRP values were significantly reduced after the administration of biological drugs overall and specifically for TNF inhibitors, but not with other molecules.

Interestingly, in the population of patients enrolled in biological treatment due to the involvement of specific areas, we observed an insignificant decrease in NLR, PLR, SII, and CRP levels (*p* > 0.05). Two explanations might be considered. First, the analyzed group was relatively small, making it challenging to reach statistical significance. Second, this group exhibited relatively low BSA and PASI scores, which has been proven in some analyses to positively correlate with CBC-derived markers. We found no existing literature on that topic in relation to psoriasis in difficult-to-treat locations. Further research is required to explore these findings.

Nowadays, to evaluate psoriasis severity, physicians implement clinical scales, with the PASI scale considered the standard. Unfortunately, it is not without drawbacks. Despite its standardization, widespread acceptance, and ability to provide a quantitative assessment, the scale has some limitations. Scoring relies on a subjective evaluation by the evaluator, and the scale is not linear, making the equivalent changes in numerical scales not comparable between mild and severe cases. Thus, CBC-derived parameters have been proposed as objective and precise tools to aid in PsO severity evaluation. We identified a positive correlation between initial NLR, PLR, and SII values and psoriasis severity assessed with BSA and PASI scores. Several studies had similar findings [5,17,18,26,33,42]. However, NLR and PLR were identified as unreliable markers of psoriasis severity [8]. Similarly, the analysis of the NHANES database showed that SII cannot be utilized as a marker of psoriasis severity [6]. Prospective studies are required for hematological biomarker validation as supportive tools in the assessment of psoriatic patients.

The associations between NLR, PLR, and SII and other clinical or demographic features of psoriatic patients remain incompletely assessed. Factors increasing overall inflammation might influence the levels of these biomarkers. On that basis, age and commodities, especially psoriatic arthritis and metabolic syndrome, may influence these parameters [42,43]. However, our group found no relationships with patient age, sex, psoriasis duration, initial CRP levels, and prior exposure to biological drugs. Interestingly, the level of inflammatory indicators may correlate with all-cause mortality in patients with psoriasis, and NMLR ((neutrophil + monocyte) ÷ lymphocyte ratio) appears to have the highest prognostic value [29]. Unfortunately, in our study, we did not analyze monocyte levels; thus, we could not calculate NMLR values. Further research is required to assess the utility of CBC-derived biomarkers in screening for patients at higher risk of comorbidities and death.

Finally, since NLR, PLR, and SII are interrelated markers with a shared inflammatory basis, we decided to investigate the relationship between the changes in their levels during the biological treatment. To perform this challenging calculation, a canonical correlation analysis was performed. Using this method, we analyzed how these multivariate datasets are related to each other and how changes in one set of variables, e.g., NLR levels, correspond to changes in the other set, e.g., PLR levels. We confirmed that the changes in these parameters occur in parallel and exhibit similar trends during psoriasis treatment. This supports the idea that, despite biological drugs targeting specific molecules, they provide a broad anti-inflammatory effect and reduce overall systemic inflammation. Importantly, this modulation of the immune response preserves the integrity between the innate and adaptive immune systems while managing psoriasis symptoms.

Our study demonstrated that CBC-derived biomarkers can effectively reflect systemic inflammation in psoriasis patients undergoing biological treatment. However, their ability to differentiate between specific biological drugs is limited, highlighting the need for more specific indicators to guide personalized therapy in psoriasis. One possible approach is cytokine profiling, specifically measuring IL-17, IL-23, IL-22, and TNF circulating levels [44,45]. Osteopontin (involved in cell recruitment and tissue remodeling) and gasdermin D (mediator of pyroptosis) also emerged as potential bioindicators [46,47]. Furthermore, specific platelet subpopulations may be linked to the severity and prognosis of infectious and autoimmune diseases [48]. Finally, proteomic analyses could offer additional insights by identifying unique protein expression patterns associated with specific biological agents [49]. While all these biomarkers are promising, they come with some limitations that must be considered when interpreting their utility. Further research is necessary to validate these markers, standardize measurement techniques, and assess their clinical utility, either individually or in combination.

While this research provides some valuable data, several limitations should be considered when interpreting its results. The data may lack consistency in measurement and documentation, which may affect the accuracy of the findings, and cannot be improved due to the retrospective nature of the analysis. The study was single-center and might not fully represent the characteristics of psoriasis patients in our country. To reach statistical significance, the drugs were grouped according to the molecules they interact with, making it impossible to identify the differences between molecules in the same class. Moreover, the number of patients in the bimekizumab subgroup was relatively low, and these patients could not be included in parts of the analysis, which could result in selection bias. Finally, while the study identifies several correlations between changes in CBC-derived biomarkers and psoriasis characteristics, causality could not be established due to the observational nature of the research. Our results should be confirmed by multicenter studies with larger sample sizes and multiple subgroups to make comparisons across all biological therapies available for psoriasis.

## 4. Materials and Methods

The medical records of 159 patients treated with biological drugs (tildrakizumab, risankizumab, guselkumab, adalimumab, infliximab, ixekizumab, secukinumab, bimekizumab, and ustekinumab) at the Dermatology Department between 1 January 2013 and 2 August 2024 were reviewed in this study. Some patients underwent more than one treatment cycle with biologics, and these cycles were analyzed as separate entities, resulting in a total of 300 treatment cycles evaluated.

**Figure 4:**
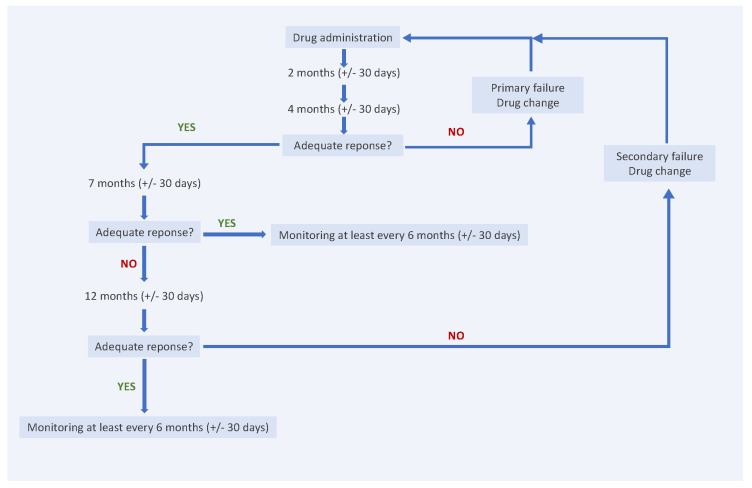
Study design.

The study employed strict inclusion and exclusion criteria in accordance with the Polish Drug Program B.47 ‘Treatment of Moderate to Severe Plaque Psoriasis (ICD-10 L40.0)’ guidelines [4]. All data were collected and analyzed retrospectively without prior knowledge of the study hypothesis. The psoriasis severity criteria in the B.47 program evolved over time, but from 2023 onwards, they have included a DLQI, BSA, and PASI score of more than 10 or the presence of psoriatic lesions in specific areas, such as the scalp, face, genital area, palms and soles, or nails, regardless of the BSA and PASI scores [4]. Patients were assessed at 2, 4, 7, 12 (with ±30-day windows), and 18 months (±90 days) following the first biologic dose (Figure 4). At each visit, DLQI, BSA, PASI, CBC, and CRP were evaluated.

To demonstrate an adequate response to treatment, patients were required to achieve a reduction in the PASI score of at least 75%, or a reduction of at least 50% accompanied by an improvement of 5 or more points on the DLQI (or CDLQI) quality of life scale. The lack of an adequate response to the administered active substance after four months (±30 days) was defined as a primary failure. The loss of adequate response observed during two consecutive visits was classified as a secondary failure (Figure 4) [4].

For the study, secondary data regarding the patients’ demographics, psoriasis severity scores (DLQI, BSA, PASI), and laboratory markers (C-reactive protein and complete blood count) were retrieved from the records. The neutrophil-to-lymphocyte ratio (NLR), platelet-to-lymphocyte ratio (PLR), and Systemic Immune-Inflammation Index (SII) values were calculated from the patients’ hemograms.

Statistical analyses were performed using Statistica 13.3 (TIBCO Software Inc., Palo Alto, CA, USA). Descriptive statistics were employed to summarize patient demographics and treatment regimens. Categorical variables were presented as counts and percentages, while continuous variables were expressed as means with ranges or medians along with IQRs depending on the distribution of the variables (normal and non-normal, respectively). Statistical analysis used Friedman’s ANOVA to assess the variability of multivariate repeated parameters, Wilcoxon analysis for bivariate repeated parameters, and Mann–Whitney ANOVA in comparisons of variability of independent characteristics. Logistic regression analysis was conducted to evaluate the effect of selected demographic and clinical variables (age, sex, disease duration, psoriasis severity) on the dependent variables. The significance level was set at *p* ≤ 0.05. To improve statistical significance, the patients were divided into 5 groups, according to the drug they had been treated with: anti-TNF (adalimumab, infliximab), anti-IL-12/23 (ustekinumab), anti-IL-17 (ixekizumab, secukinumab), anti-IL-23 (guselkumab, risankizumab, tildrakizumab), and anti-IL-17AF (bimekizumab). ANOVA for repeated measures for NLR, PLR, and SII could not be performed for the anti-IL17AF group due to the insufficient amount of data. A canonical analysis was used to perform a multivariate analysis of the relationship of multiple repeated measures for both the dependent and independent variables, expressed on a nominal scale. In this manner, intercorrelations between inflammatory markers, i.e., NPL, PLR, and SII, were compared. Each of these measurements was taken a total of six times over the course of the observation and became, successively, the dependent or independent variable during the analysis.

The study was exempted from the requirement for bioethics committee approval due to its retrospective nature, involving only the analysis of previously collected data (the Bioethics Committee of the Medical University of Lodz decision nr RNN/196/24/KE from 10 September 2024). The study was performed in accordance with the Helsinki Declaration of 1964 and its later amendments. Data collection and handling complied with applicable laws, regulations, and guidance regarding patient protection, including patient privacy.

## 5. Conclusions

Biomarkers derived from routine blood tests, including the neutrophil-to-lymphocyte ratio (NLR), platelet-to-lymphocyte ratio (PLR), and Systemic Immune-Inflammation Index (SII), represent a promising, widely available, easy-to-measure, and objective tool in psoriasis evaluation. However, their relationship with psoriasis severity and the presence of comorbidities remains incompletely understood. While they show potential as supportive tools for monitoring treatment effectiveness, their utility is limited due to a lack of established prognostic values and cut-off points. Prospective studies are required to clarify their clinical significance and to refine their application in routine practice.

## Figures and Tables

**Table 2:** Patient characteristics (*N* = 159). PsO—psoriasis.

Variable	Value
Total	159
Males (%), Females (%)	90 (56.60%), 69 (43.40%)
Bio-naïve	130 (81.76%)
Previous exposure to biological treatment	29 (18.24%)
Mean age of PsO onset	25.73 ± 13.57 years
Mean duration of PsO prior biological treatment	18.61 ± 12.96 years
Mean age at the biological treatment commencement	44.35 ± 13.94 years
Single cycle of biological treatment	100 (62.89%)
Multiple cycles of biological treatment	59 (37.11%)
Mean DLQI	19.38 ± 6.06
Mean BSA	23.08 ± 15.2
Mean PASI	17.39 ± 8.24

## Data Availability

The data presented in this study are available on request from the corresponding author due to legal reasons.

## Supplementary Materials

The following supporting information can be downloaded at: https://www.mdpi.com/article/10.3390/ijms26083868/s1.

## Abbreviations

The following abbreviations are used in this manuscript:

PsO	psoriasis
Th1	T-helper 1 lymphocytes
IL	interleukin
IL-17	interleukin 17
IL-17A	subunit A of interleukin 17
IL-17AF	subunit A and F of interleukin 17
IL-23	interleukin 23
IL-12/23	interleukin 12 and 23
TNF	tumour necrosis factor-alpha
Anti-IL-17	interleukin 17 antagonist
Anti-IL-23	interleukin 23 antagonist
Anti-IL-12/23	interleukin 12 and 23 antagonist
Anti-IL-17A	antagonist of subunit A of interleukin 17
Anti-IL-17AF	antagonist of subunit A and F of interleukin 17
Anti-TNF	tumor necrosis factor inhibitor
DLQI	Dermatology Life Quality Index
BSA	Body Surface Area
BSA0	baseline Body Surface Area
PASI	Psoriasis Area and Severity Index
PASI0	baseline Psoriasis Area and Severity Index
NLR	neutrophil-to-lymphocyte ratio
PLR	platelet-to-lymphocyte ratio
SII	Systemic Immune-Inflammation Index
NMR	neutrophil-to-monocyte ratio
MLR	monocyte-to-lymphocyte ratio
NMLR	ratio of the sum of neutrophil and monocyte, to lymphocyte
CRP	C-reactive protein
CBC	complete blood count
*p*	*p*-value
r	correlation coefficient
ANOVA	analysis of variance
Cl	confidence interval

## References

[1] Yamazaki F. (2021). Psoriasis: Comorbidities. J. Dermatol..

[2] Rendon A., Schäkel K. (2019). Psoriasis Pathogenesis and Treatment. Int. J. Mol. Sci..

[3] Reich A., Szepietowski J., Adamski Z., Chodorowska G., Kaszuba A., Krasowska D., Lesiak A., Maj J., Narbutt J., Osmola-Mankowska A. (2018). Psoriasis. Diagnostic and Therapeutic Recommendations of the Polish Dermatological Society. Part II: Moderate to Severe Psoriasis. Dermatol. Rev. Przegląd Dermatol..

[4] Kimak-Pielas A., Robak E., Zajdel R., Zebrowska A. (2024). Demographics, Disease Characteristics, and Treatment Patterns of Patients with Plaque Psoriasis Treated with Biological Drugs: The Experience of a Single-Centre Study in Poland. J. Clin. Med..

[5] Liu Y.C., Chuang S.H., Chen Y.P., Shih Y.H. (2024). Associations of Novel Complete Blood Count-Derived Inflammatory Markers with Psoriasis: A Systematic Review and Meta-Analysis. Arch. Dermatol. Res..

[6] Guo H.H., Chen R.X. (2024). Association of Systemic Inflammation Index with Psoriasis Risk and Psoriasis Severity: A Retrospective Cohort Study of NHANES 2009 to 2014. Medicine.

[7] Menzel A., Samouda H., Dohet F., Loap S., Ellulu M.S., Bohn T. (2021). Common and Novel Markers for Measuring Inflammation and Oxidative Stress Ex Vivo in Research and Clinical Practice—Which to Use Regarding Disease Outcomes?. Antioxidants.

[8] Ye J.H., Zhang Y., Naidoo K., Ye S. (2024). Neutrophil-to-Lymphocyte Ratio and Platelet-to-Lymphocyte Ratio in Psoriasis: A Systematic Review and Meta-Analysis. Arch. Dermatol. Res..

[9] Fei Y., Wang X., Zhang H., Huang M., Chen X., Zhang C. (2020). Reference Intervals of Systemic Immune-Inflammation Index, Neutrophil to Lymphocyte Ratio, Platelet to Lymphocyte Ratio, Mean Platelet Volume to Platelet Ratio, Mean Platelet Volume and Red Blood Cell Distribution Width-Standard Deviation in Healthy Han Adults in Wuhan Region in Central China. Scand. J. Clin. Lab. Investig..

[10] Kosidło J.W., Wolszczak-Biedrzycka B., Dymicka-Piekarska V., Dorf J., Matowicka-Karna J. (2023). Clinical Significance and Diagnostic Utility of NLR, LMR, PLR and SII in the Course of COVID-19: A Literature Review. J. Inflamm. Res..

[11] Misiewicz A., Dymicka-Piekarska V. (2023). Fashionable, but What Is Their Real Clinical Usefulness? NLR, LMR, and PLR as a Promising Indicator in Colorectal Cancer Prognosis: A Systematic Review. J. Inflamm. Res..

[12] Tamaki S., Nagai Y., Shutta R., Masuda D., Yamashita S., Seo M., Yamada T., Nakagawa A., Yasumura Y., Nakagawa Y. (2023). Combination of Neutrophil-to-Lymphocyte and Platelet-to-Lymphocyte Ratios as a Novel Predictor of Cardiac Death in Patients With Acute Decompensated Heart Failure With Preserved Left Ventricular Ejection Fraction: A Multicenter Study. J. Am. Heart Assoc..

[13] Mangoni A.A., Zinellu A. (2024). Diagnostic Accuracy of the Neutrophil-to-Lymphocyte Ratio and the Platelet-to-Lymphocyte Ratio in Rheumatoid Arthritis: A Systematic Review and Meta-Analysis. Clin. Exp. Med..

[14] Tangjitgamol S., Udayachalerm W., Wanishsawad C., Kaewwanna W., Ativanichayapong N. (2024). Association of Neutrophil-to-Lymphocyte Ratio and Platelet-to-Lymphocyte Ratio and Coronary Artery Disease Among the Physicians. J. Inflamm. Res..

[15] Zinellu A., Paliogiannis P., Mangoni A.A. (2024). A Systematic Review and Meta-Analysis of the Diagnostic Accuracy of the Neutrophil-to-Lymphocyte Ratio and the Platelet-to-Lymphocyte Ratio in Systemic Lupus Erythematosus. Clin. Exp. Med..

[16] Paliogiannis P., Satta R., Deligia G., Farina G., Bassu S., Mangoni A.A., Carru C., Zinellu A. (2019). Associations between the Neutrophil-to-Lymphocyte and the Platelet-to-Lymphocyte Ratios and the Presence and Severity of Psoriasis: A Systematic Review and Meta-Analysis. Clin. Exp. Med..

[17] Yorulmaz A., Hayran Y., Akpinar U., Yalcin B. (2020). Systemic Immune-Inflammation Index (SII) Predicts Increased Severity in Psoriasis and Psoriatic Arthritis. Curr. Health Sci. J..

[18] Melikoglu M., Pala E. (2023). Systemic Immune-Inflammation Index as a Biomarker of Psoriasis Severity. Arch. Basic. Clin. Res..

[19] Zhao X., Li J., Li X. (2024). Association between Systemic Immune-Inflammation Index and Psoriasis: A Population-Based Study. Front. Immunol..

[20] Hong J., Lian N., Li M. (2023). Association between the Neutrophil-to-Lymphocyte Ratio and Psoriasis: A Cross-Sectional Study of the National Health and Nutrition Examination Survey 2011–2014. BMJ Open.

[21] Nguyen H.T., Vo L.D.H., Pham N.N. (2022). Neutrophil-to-Lymphocyte and Platelet-to-Lymphocyte Ratios as Inflammatory Markers in Psoriasis: A Case-Control Study. Dermatol. Rep..

[22] Tiucă O.M., Morariu S.H., Mariean C.R., Tiucă R.A., Nicolescu A.C., Cotoi O.S. (2024). Impact of Blood-Count-Derived Inflammatory Markers in Psoriatic Disease Progression. Life.

[23] Yurtdaş M., Yaylali Y.T., Kaya Y., Özdemir M., Özkan I., Aladaʇ N. (2014). Neutrophil-to-Lymphocyte Ratio May Predict Subclinical Atherosclerosis in Patients with Psoriasis. Echocardiography.

[24] Vega M., Sanchez C., Hernandez S., Pulido N., Castro Z. (2024). 52028 Prediction of Psoriatic Arthritis in Mexican Patients with Psoriasis: Utility of the Neutrophil-to-Lymphocyte Ratio. J. Am. Acad. Dermatol..

[25] Hammad R., Hamdino M., El-Nasser A.M. (2020). Role of Neutrophil-to-Lymphocyte Ratio, Platelet-to-Lymphocyte Ratio, Mean Platelet Volume in Egyptian Patients with Psoriasis Vulgaris. Egypt J. Immunol..

[26] Solak B., Özta¸s R., Kara Ö., Ana D., Dalı B., Fakültesi T. (2024). Assessing Systemic Inflammatory Markers in Psoriasis: A Retrospective Study. Trop. Med. Int. Health.

[27] Li H., Zhang H., Zhao X., Huang J., Zhang J., Liu Z., Wen J., Qin S. (2024). The Role of C-Reactive Protein and Genetic Predisposition in the Risk of Psoriasis: Results from a National Prospective Cohort. BMC Rheumatol..

[28] Albayrak H. (2023). Neutrophil-to-Lymphocyte Ratio, Neutrophil-to-Monocyte Ratio, Platelet-to-Lymphocyte Ratio, and Systemic Immune-Inflammation Index in Psoriasis Patients: Response to Treatment with Biological Drugs. J. Clin. Med..

[29] Zhao Y., Yang X.T., Bai Y.P., Li L.F. (2023). Association of Complete Blood Cell Count-Derived Inflammatory Biomarkers with Psoriasis and Mortality. Clin. Cosmet. Investig. Dermatol..

[30] Asahina A., Kubo N., Umezawa Y., Honda H., Yanaba K., Nakagawa H. (2017). Neutrophil-Lymphocyte Ratio, Platelet-Lymphocyte Ratio and Mean Platelet Volume in Japanese Patients with Psoriasis and Psoriatic Arthritis: Response to Therapy with Biologics. J. Dermatol..

[31] An I., Ucmak D., Ozturk M. (2020). The Effect of Biological Agent Treatment on Neutrophil-to-Lymphocyte Ratio, Platelet-to-Lymphocyte Ratio, Mean Platelet Volume, and C-Reactive Protein in Psoriasis Patients. Postepy Dermatol. Alergol..

[32] Kulakli S., Oguz I., Aksan B. (2023). Could Blood Cell-Based Inflammatory Markers Be Used to Monitor Response to Biologic Therapy in Psoriasis?. Sisli Etfal Hastan. Tıp Bul..

[33] Şener G., İnan Yuksel E., Gökdeniz O., Karaman K., Canat H.D. (2023). The Relationship of Hematological Parameters and C-Reactive Protein (CRP) With Disease Presence, Severity, and Response to Systemic Therapy in Patients With Psoriasis. Cureus.

[34] Morariu S.H., Cotoi O.S., Tiucă O.M., Baican A., Gheucă-Solovăstru L., Decean H., Brihan I., Silaghi K., Biro V., Șerban-Pescar D. (2024). Blood-Count-Derived Inflammatory Markers as Predictors of Response to Biologics and Small-Molecule Inhibitors in Psoriasis: A Multicenter Study. J. Clin. Med..

[35] Asahina A., Umezawa Y., Yanaba K., Nakagawa H. (2016). Serum C-Reactive Protein Levels in Japanese Patients with Psoriasis and Psoriatic Arthritis: Long-Term Differential Effects of Biologics. J. Dermatol..

[36] Vadakayil A.R., Dandekeri S., Kambil S.M., Ali N.M. (2015). Role of C-Reactive Protein as a Marker of Disease Severity and Cardiovascular Risk in Patients with Psoriasis. Indian. Dermatol. Online J..

[37] Beygi S., Lajevardi V., Abedini R. (2014). C-Reactive Protein in Psoriasis: A Review of the Literature. J. Eur. Acad. Dermatol. Venereol..

[38] Coimbra S., Oliveira H., Reis F., Belo L., Rocha S., Quintanilha A., Figueiredo A., Teixeira F., Castro E., Rocha-Pereira P. (2010). C-Reactive Protein and Leucocyte Activation in Psoriasis Vulgaris According to Severity and Therapy. J. Eur. Acad. Dermatol. Venereol..

[39] Hoffmann J.H.O., Knoop C., Schäkel K., Enk A.H., Hadaschik E.N. (2021). Evaluation of Psoriasis Area and Severity Index as a Proxy for Bio-Markers of Systemic Disease under Treatment with Tumour Necrosis Factor-Alpha and Interleukin 12/23 Antagonists in Patients with Psoriasis: A Retrospective Cohort Study of 186 Treatment Cycles. Acta Derm. Venereol..

[40] Strober B., Teller C., Yamauchi P., Miller J.L., Hooper M., Yang Y.C., Dann F. (2008). Effects of Etanercept on C-Reactive Protein Levels in Psoriasis and Psoriatic Arthritis. Br. J. Dermatol..

[41] Strober B.E., Poulin Y., Teller C., Wang Y., Williams D.A., Goldblum O.M. (2014). Changes in C-Reactive Protein in Patients with Moderate-to-Severe Psoriasis Switched to Adalimumab Therapy after Suboptimal Response to Etanercept, Methotrexate or Phototherapy. J. Eur. Acad. Dermatol. Venereol..

[42] Kommoss K.S., Bieler T., Ringen J., Lehmann A., Mihalceanu S., Hobohm L., Keller K., Brand A., Fischer B., Kramer D. (2024). A Simple Tool for Evaluation of Inflammation in Psoriasis: Neutrophil-to-Lymphocyte and Platelet-to-Lymphocyte Ratio as Markers in Psoriasis Patients and Related Murine Models of Psoriasis-like Skin Disease. J. Mol. Med..

[43] Rodríguez-Cerdeira C., Cordeiro-Rodríguez M., Carnero-Gregorio M., López-Barcenas A., Martínez-Herrera E., Fabbrocini G., Sinani A., Arenas-Guzmán R., González-Cespón J.L. (2019). Biomarkers of Inflammation in Obesity-Psoriatic Patients. Mediat. Inflamm..

[44] Solberg S.M., Sandvik L.F., Eidsheim M., Jonsson R., Bryceson Y.T., Appel S. (2018). Serum Cytokine Measurements and Biological Therapy of Psoriasis–Prospects for Personalized Treatment?. Scand. J. Immunol..

[45] Florian T.L., Florian I.A., Vesa S.C., Beni L., Orăsan M. (2024). Inflammatory Cytokines and Clinical Outcome Following Biological Therapy in Adult Bio-Naïve Psoriasis Patients. Curr. Issues Mol. Biol..

[46] Lian N., Chen Y., Chen S., Zhang Y., Chen H., Yang Y., Gu H., Chen Q., Li M., Chen X. (2023). Gasdermin D-Mediated Keratinocyte Pyroptosis as a Key Step in Psoriasis Pathogenesis. Cell Death Dis..

[47] Kimak A., Woźniacka A. (2024). The Role of Osteopontin in Psoriasis-A Scoping Review. J. Clin. Med..

[48] Qiu X., Nair M.G., Jaroszewski L., Godzik A. (2024). Deciphering Abnormal Platelet Subpopulations in COVID-19, Sepsis and Systemic Lupus Erythematosus through Machine Learning and Single-Cell Transcriptomics. Int. J. Mol. Sci..

[49] Radulska A., Pelikant-Małecka I., Jendernalik K., Dobrucki I.T., Kalinowski L. (2023). Proteomic and Metabolomic Changes in Psoriasis Preclinical and Clinical Aspects. Int. J. Mol. Sci..

