# Supplementary material for: The Relationship Between Neutrophil-to-Lymphocyte Ratio, Platelet-to-Lymphocyte Ratio, and Systemic Immune-Inflammation Index Markers and Response to Biological Therapy in Patients with Psoriasis
Source: Int J Mol Sci. 2025 Apr 19;26(8):3868. doi: 10.3390/ijms26083868 (PMC12028229; doi:10.3390/ijms26083868)
Supplement: Supplementary file 1 [file ijms-26-03868-s001.zip › ijms-3572143-supplementary.pdf]

Table S1. NLR, PLR, SII, and CRP (mg/l) values during the observation for each biological drug and patients with special areas enrollment (mean ± SD).

	Total	Anti-TNF	Anti-IL-23	Anti-IL-17	Anti-IL-12/23	Special areas
			NLR			
NLR 0	2.3380±1.3565	2.0147±0.6647	2.0219±0.5151	2.3358±0.8185	3.0635±2.5289	2.3667±1.5102
NLR 1	1.7828±0.8967	1.5813±0.7093	1.8010±0.8591	1.7190±0.5148	2.1706±1.4373	1.3667±0.5323
NLR 2	1.7975±0.7820	1.6972±0.8597	1.9161±1.1050	1.7556±0.5682	1.9334±0.7642	1.4333±0.6932
NLR 3	1.6916±0.7048	1.7019±0.8762	1.6641±0.7793	1.7586±0.5996	1.6166±0.5708	1.3000±0.6708
NLR 4	1.8507±0.7502	1.7666±0.9583	2.2678±0.8575	1.8312±0.6355	1.7489±0.3570	1.9000±0.5477
NLR 5	1.8153±0.7590	1.6895±0.9537	1.8531±0.9336	1.9620±0.4667	1.8308±0.6587	1.5667±0.6625
			PLR			
PLR 0	146.0028±59.2079	132.5694±58.2937	131.2030±35.2669	154.5775±58.3101	167.4354±74.1218	149.6667±97.4347
PLR 1	124.0640±54.2781	108.7970±52.3405	129.2657±48.6626	133.4197±54.2821	135.0966±64.7214	109.7333±38.4624
PLR 2	127.1274±50.7434	114.2272±48.2841	122.1345±51.3475	134.6107±48.6916	142.7924±60.1210	107.8667±33.2836
PLR 3	122.4475±53.1906	112.1009±65.0766	118.0600±39.6026	130.0986±40.2293	133.3954±57.5453	105.3000±39.3405
PLR 4	125.8029±42.6646	105.1413±38.4193	149.1929±48.2127	137.9368±37.4657	131.1085±44.4233	129.3000±38.5496

PLR 5	124.9965±44.0053	104.8719±45.3769	146.0934±29.7148	135.1553±36.5425	133.0847±50.6725	118.9000±33.1368
			SII			
SII 0	593.6625±389.9643	513.7207±295.3777	475.2875±165.0264	598.3759±250.6401	792.2496±659.3442	640.9667±540.2927
SII 1	423.8593±252.6832	378.6896±212.2216	421.0520±283.3226	418.8794±126.2664	504.6173±396.9183	361.2667±193.5141
SII 2	426.2561±234.3299	417.6544±284.9486	440.8943±334.5321	418.6811±153.0802	439.6068±190.3075	350.4333±234.7758
SII 3	412.3682±237.9478	415.9790±280.5970	388.1058±274.2837	436.6428±178.1530	394.3558±243.3074	325.4000±229.6624
SII 4	451.8039±217.6875	429.5981±248.0401	553.0342±299.7040	464.4090±157.1238	410.5713±235.6551	514.6000±238.6919
SII 5	453.8275±251.4009	420.4154±308.8989	485.7786±321.3222	496.1221±143.6192	440.5713±235.6551	417.2000±242.2833
			CRP			
CRP 0	5.6571±6.8202	5.1923±6.4576	6.1000±3.2272	4.2778±3.1324	7.6875±11.3834	3.8173 ±9.0144
CRP 1	2.6657±2.8359	2.1769±2.4393	2.9800±3.4252	3.1000±2.1395	2.7750±4.0195	5.2091 10.0310
CRP 2	2.9029±3.8515	2.5846±3.4633	2.3200±2.5154	3.0000±1.9805	3.6750±6.5012	2.8333 2.5884
CRP 3	2.4543±2.1147	2.7538±2.6485	2.1400±1.8730	2.9779±1.9331	1.5750±1.3957	3.4333 3.6144
CRP 4	2.8857±2.9738	3.4692±4.1323	2.8600±3.0138	3.0667±1.8808	1.7500±1.5166	2.9080 2.5665
CRP 5	2.5600±2.3651	2.5462±2.2773	2.2800±2.4743	3.2000±3.2787	2.0375±1.2648	3.3889 3.8541

Table S2. p=values for NLR, PLR, SII, and CRP changes for each biological drug (ANOVA).

	Anti-TNF	Anti-IL-23	Anti-IL-17	Anti-IL-12/23
NLR	0.17168	0.41588	0.33979	0.56873
PLR	0.01763	0.86327	0.32305	0.54528
SII	0.03680	0.37549	0.01455	0.34229
CRP	0.01809	0.25154	0.20204	0.44183

Table S3. p=values for NLR, PLR, SII, and CRP comparison between biological drugs (ANOVA). A. NLR, B. PLR, C. SII, D. CRP.

A. NLR

	Anti-TNF	Anti-IL-12/23	Anti-IL-23	Anti-IL-17
Anti-TNF	-	0.115827	0.722893	0.875185
Anti-IL-12/23	0.115827	-	0.328711	0.427561
Anti-IL-23	0.722893	0.328711	-	0.383914
Anti-IL-17	0.875185	0.427561	0.383914	-

B. PLR

	Anti-TNF	Anti-IL-12/23	Anti-IL-23	Anti-IL-17
Anti-TNF	-	0.965542	0.016956	0.825270
Anti-IL-12/23	0.965542	-	0.076530	0.848548
Anti-IL-23	0.016956	0.076530	-	0.243550
Anti-IL-17	0.825270	0.848548	0.243550	-

C. SII

	Anti-TNF	Anti-IL-12/23	Anti-IL-23	Anti-IL-17
Anti-TNF	-	0.093848	0.580357	0.872445
Anti-IL-12/23	0.093848	-	0.200453	0.366010
Anti-IL-23	0.580357	0.200453	-	0.416964
Anti-IL-17	0.872445	0.366010	0.416964	-

D. CRP

	Anti-TNF	Anti-IL-12/23	Anti-IL-23	Anti-IL-17
Anti-TNF	-	0.622358	0.955678	0.860146
Anti-IL-12/23	0.622358	-	0.969686	0.448719
Anti-IL-23	0.955678	0.969686	-	0.210241
Anti-IL-17	0.860146	0.448719	0.210241	-

Table S4. Canonical analysis of NLR, PLR, and SII changes correlations. A. NLR vs. PLR, B. NLR vs. SII, C. PLR vs. SII.

A.

	Canonical variables					
Variables of one set	U1	U2	U3	U4	U5	U6
0 NLR	-1.418	-0.199	0.1523	-0.128	0.305	0.340
1 NLR	0.936	1.049	0.4909	0.601	0.153	0.112
2 NLR	-0.358	-0.484	-0.2268	0.864	1.015	-1.226
3 NLR	0.048	-0.332	-0.8526	1.181	0.210	1.668
4 NLR	0.110	-0.020	0.0047	-0.836	1.014	0.560

5 NLR	0.151	0.774	-0.1808	-1.659	-1.996	-1.235
Canonical correlations r_c/p	0.9251	0.8514	0.7616	0.6904	0.5044	0.0467
	0.0000	0.0000	0.0000	0.0021	0.0868	0.8062
Total redundancy			62.31%			
Second-order redundancies	3.96%	40.70%	14.75%	2.36%	0.52%	0.015%

	Canonical variables					
Variables of the second set	V1	V2	V3	V4	V5	V6
0 PLR	-1.779	0.283	0.428	-0.081	0.819	0.171
1 PLR	0.565	1.412	1.492	0.954	-1.154	0.327
2 PLR	0.411	-0.997	-0.895	1.060	0.239	-1.996
3 PLR	0.851	0.029	-1.556	0.044	0.444	2.467
4 PLR	0.735	0.180	-0.154	-1.022	2.282	0.384
5 PLR	-0.953	-0.027	0.150	-0.968	-2.414	-1.609

B.

	Canonical variables					
Variables of one set	U1	U2	U3	U4	U5	U6
0 NLR	-0.4945	-0.1795	-1.325	-0.241	-0.159	-0.428
1 NLR	-0.7310	0.2302	1.344	-0.253	0.396	-0.022
2 NLR	0.5456	-0.1894	0.145	-1.167	-1.212	-0.713
3 NLR	0.1254	-0.4172	0.107	0.684	0.962	-1.861
4 NLR	0.2535	0.6981	-0.519	-0.636	0.909	-0.024

5 NLR	0.0630	-0.8155	0.007	0.740	-0.257	2.763
Canonical correlations r_c/p	0.9923	0.9731	0.9622	0.9485	0.9083	0.8888
	0.0000	0.0000	0.0000	0.0000	0.0000	0.0000
Total redundancy			91.30%			
Second-order redundancies	11.46%	41.83%	4.69%	13.51%	18.01%	1.79%

	Canonical variables					
Variables of the second set	V1	V2	V3	V4	V5	V6
0 SII	-0.5074	-0.2372	-1.377	-0.369	0.040	-0.415
1 SII	-0.8899	0.1904	1.458	-0.168	0.118	0.190
2 SII	0.7851	-0.1819	0.370	-1.674	-1.206	-0.737
3 SII	0.0285	-0.4524	-0.135	1.241	1.108	-1.825
4 SII	0.3026	0.7027	-0.544	-0.693	0.982	0.039
5 SII	0.1142	-0.7417	0.100	0.967	-0.327	2.671

C.

	Canonical variables					
Variables of one set	U1	U2	U3	U4	U5	U6
0 PLR	1.6487	-0.4848	-0.456	0.788	0.593	-0.002
1 PLR	-0.5673	0.5033	-2.209	-0.170	-1.060	0.530
2 PLR	-0.4061	-0.1179	0.632	-1.022	0.129	-2.342
3 PLR	-0.1527	0.4726	0.932	-1.437	1.097	2.247
4 PLR	-0.6009	0.3299	0.535	0.938	2.282	0.394

5 PLR	0.5215	0.2528	0.511	0.927	-2.799	-0.997
Canonical correlations r_c/p	0.9465	0.8533	0.8211	0.7589	0.5001	0.0820
	0.0000	0.0000	0.000	0.0002	0.0880	0.6666
Total redundancy			70.07%			
Second-order redundancies	17.45%	40.38%	6.93%	3.99	1.30	0.00
			Canonical variables			
Variables of the second set	V1	V2	V3	V4	V5	V6
0 SII	1.3457	-0.4386	0.041	0.459	0.367	0.410
1 SII	-0.7802	0.8088	-1.309	-0.168	-0.162	-0.043
2 SII	0.2918	-0.3420	-0.253	-0.811	1.414	-1.632
3 SII	0.3071	-0.2041	0.383	-1.482	-0.056	1.960
4 SII	-0.1529	0.1660	0.469	0.768	1.094	0.520
5 SII	-0.3370	0.9195	0.757	1.203	-2.163	-1.046
